# Development of the Ion Exchange-Gravimetric Method for Sodium in Serum as a Definitive Method

**DOI:** 10.6028/jres.101.017

**Published:** 1996

**Authors:** John R. Moody, Thomas W. Vetter

**Affiliations:** National Institute of Standards and Technology, Gaithersburg, MD 20899-0001

**Keywords:** accuracy, definitive method, gravimetry, human serum, instrumental determination, ion-exchange, NCCLS, repeatability, sodium, uncertainty

## Abstract

An ion exchange-gravimetric method, previously developed as a National Committee for Clinical Laboratory Standards (NCCLS) reference method for the determination of sodium in human serum, has been re-evaluated and improved. Sources of analytical error in this method have been examined more critically and the overall uncertainties decreased. Additionally, greater accuracy and repeatability have been achieved by the application of this definitive method to a sodium chloride reference material. In this method sodium in serum is ion-exchanged, selectively eluted and converted to a weighable precipitate as Na_2_SO_4_. Traces of sodium eluting before or after the main fraction, and precipitate contaminants are determined instrumentally. Co-precipitating contaminants contribute less than 0.1 % while the analyte lost to other eluted ion-exchange fractions contributes less than 0.02 % to the total precipitate mass. With improvements, the relative expanded uncertainty (*k* = 2) of the method, as applied to serum, is 0.3 % to 0.4 % and is less than 0.1 % when applied to a sodium chloride reference material.

## 1. Introduction

The accurate determination of constituents in body fluids is a fundamental function of most clinical laboratories. The corroboration of the validity of the data produced may be accomplished through the use of an authentic reference material, the use of an established measurement protocol, or both. NCCLS and NIST collaborated some years ago to create an accurate basis for clinical measurements of serum for the elements Na, K, Li, Ca, Mg, and Cl. This collaboration involved the production of a series of clinical standard reference materials (SRMs) and a series of reference methods [1–5]. The reference methods, which are elaborate protocols for FAAS (flame atomic absorption spectrometry) and FAES (flame atomic emission spectrometry) methods, were validated in turn through comparison to definitive methods developed at NIST.

We describe here a re-evaluation of the definitive method developed for sodium in serum during this collaboration. In this context, definitive methods are defined in the NCCLS document NRSCL1-A entitled “Development of Definitive Methods for the National Reference System for the Clinical Laboratory” [6]. Regardless of application, definitive methods generally are considered as being highly accurate methods in which all identifiable sources of systematic error have been evaluated. The definitive method is usually quite different from the analytical techniques used in the clinical working methods. Gravimetry and isotope dilution mass spectrometry are two techniques used for definitive methods at NIST. Since sodium has only one stable isotope, gravimetry was the technique of choice.

The ion exchange-gravimetric procedure for sodium was based in part upon a classical procedure for sodium [7] and in part upon the ion exchange properties of sodium. The Na in serum is ion-exchanged onto a large column of ion exchange resin. By a series of washings and selective elution, greater than 99.9 % of the Na can be eluted and collected. The Na is converted to a weighable form as Na_2_SO_4_ by the addition of sulfuric acid and subsequent ignitions.

In this paper we re-evaluate the ion exchange-gravimetric procedure, previously developed to evaluate the flame atomic emission spectrometry (FAES) reference method for sodium [2], and other issues related to the definitive method for the determination of sodium in serum. This re-evaluation was made possible by improvements in the procedure and the study of what is now about a 15 year database of analyses by the definitive method. As a result, the evaluation of sources of analytical error can be examined more critically than before and the overall uncertainties improved.

In addition, one deficiency in prior work was the inability to establish the independence of the method with respect to the operator. Now, we can be confident that any chemist with the requisite skill and equipment can reproduce this definitive method. Finally, by application of this definitive method to reference materials other than serum, it is found that the basic accuracy of the method is much better than when applied to serum samples. Results for SRM 919a, sodium chloride, allowed us to define further what we believe to be the major sources of error in the definitive method for sodium in serum. The definitive method most recently has been applied to the analysis of Na in SRM 956 and SRM 909a, the newest NIST serum standard reference materials.

## 2. Materials and Methods

**Fig. 1:**
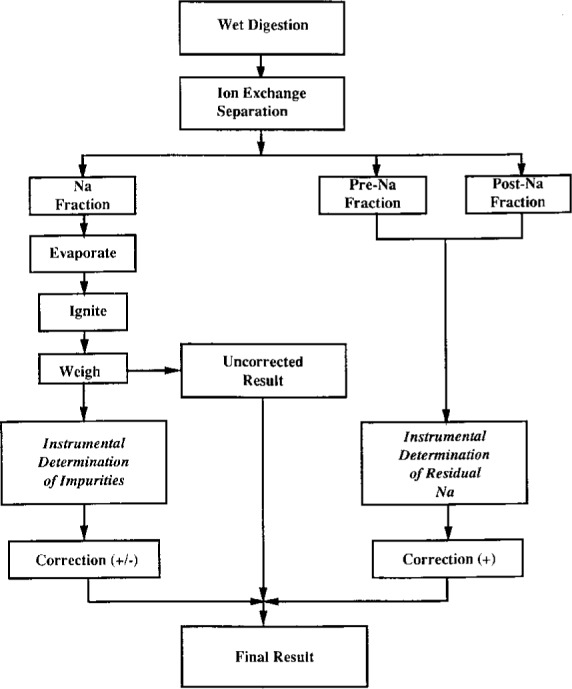
Ion-exchange-gravimetric determination of sodium.

All acids were purified by a sub-boiling distillation technique, from quartz stills [8, 9]. All water was deionized and distilled. A flow chart of the steps involved in this method is presented in Fig. 1.

### 2.1 Sampling

Samples of frozen but freshly thawed human serum (SRM 956), reconstituted freeze-dried human serum (SRMs 909 and 909a) and samples from standard solutions of sodium chloride (SRM 919 and 919a) containing 8 mg to 70 mg of sodium were taken (The range of the equivalent amount of sodium in specific samples was SRM 956, 8 mg to 11 mg; SRMs 909 and 909a, 29 mg to 35 mg, and SRMs 919 and 919a, 34 mg to 70 mg). A small excess of sample was drawn into a clean plastic syringe or disposable pipette and weighed. After the sample was transferred to a clean Teflon^1^ fluorinated ethylene propylene (FEP) beaker, the syringe or pipette was reweighed. Serum samples either were wet-digested or diluted to a 30 mL volume and then added directly onto the ion exchange column.

### 2.2 Wet Digestion

Serum samples were wet-digested in a Teflon FEP beaker in a mixture of 10 g water, 7 g HNO_3_ and 5 g HClO_4_ while covered with a Teflon lid. Digestion temperatures were limited by the melting point of Teflon FEP. After digestion, the samples were evaporated to near dryness to remove most of the HClO_4_. The residues were dissolved in 30 mL of water with gentle heating (~ 50 °C) to promote dissolution and allowed to cool.

### 2.3 Ion exchange Separation

The cooled sample solutions were poured carefully, 5 mL at a time, into polycarbonate columns (0.9 cm inside diameter, 48 cm high) containing AG 50W-X8 (100 to 200 mesh) cation exchange resin (30 cm by 0.9 cm bed volume) which had been pretreated with ~ 6 mol/L HCl and then rinsed with distilled water until the effluent had a pH 5. Occasionally, the addition of serum samples that had not been wet-digested resulted in the formation of a protein cake of material at the top of the column. When necessary, this cake was stirred up with a Teflon stirring rod to prevent restriction of the effluent flow rate. The columns were eluted with ~ 0.4 mol/L HCl. The normal order of elution of cations with this reagent is Li^+^, Na^+^, K^+^, Mg^++^, and Ca^++^. Since there is ordinarily very little Li in serum, the separation of sodium from lithium occurs readily. Because the efficiency of the chromatography may vary slightly, it was very important to use a flame test in judging when to collect the various fractions. Confirmatory flame tests for Li, Na, and K on the edges of the sodium fraction (where the sodium concentration is low) were made. Thus, the 10 mL to 50 mL fractions just before and after the sodium fraction were examined by FAES to determine the presence of sodium, lithium, and potassium. If necessary, suitable corrections were made. Details of the separation procedure are given in NBS Special Publication 260–60 [2].

### 2.4 Evaporation, Ignition, and Weighing of Sodium Sulfate

The sodium fraction from each column was collected in a 100 mL Teflon FEP beaker, 1 mL of dilute H_2_SO_4_ (containing at least 3 times more moles of sulfate than the expected moles of sodium) was added, and the sample was evaporated to a volume of ~ 8 mL. Each concentrate was transferred quantitatively to a clean platinum crucible and gently evaporated to dryness on a hot plate. Then ~ 0.3 g of (NH_4_)_2_CO_3_ powder was added to minimize formation of sodium bisulfate and convert any sodium bisulfate to sodium sulfate. The platinum crucible was covered, heated to 800 °C, held at 800 °C for 1 h, then rapidly heated to 900 °C for 20 min to complete the transformation to Na_2_SO_4_. The time at 900 °C is limited to minimize volatilization of Na_2_SO_4_.

The sample was weighed after it had cooled overnight in a desiccator. Then, three drops of distilled water were added to wet the sample and the water was evaporated off at < 90 °C. The sample was heated again to 800 °C, then ignited at 900 °C for 20 min, and finally cooled in a desiccator overnight and reweighed. If the difference in mass was less than 20 μg, constant mass was assumed. Usually, only two ignitions were required. After the mean mass of the blanks was subtracted, the difference in mass between the sample and platinum crucible was taken as the mass of Na_2_SO_4_. The mass of Na_2_SO_4_ divided by the gravimetric factor of 3.0893 gives the mass of sodium [10]. More details of this procedure are given in NBS Special Publication 260–60 [2].

## 3. The Quantification of Sources of Systematic Error and Uncertainty

All identifiable sources of systematic error and uncertainty in the definitive method for Na in serum have been investigated. Some may find this study of quantitative transfer and mechanical losses to be trivial, but no presumptions about quantification were made. Although some components of uncertainty have been found to be negligible, all processes have been evaluated for their contribution to the uncertainty of the definitive method. The topics addressed are: sample handling, density, sample mass, sample volume, ion exchange chromatography, the influence of sample form on ion exchange, blanks, ignition and weighing of samples, and recovery of standards. All uncertainty components are calculated using the CIPM approach [11].

### 3.1 Sample Handling

The most likely source of systematic error would be losses in collection and transfer. There are three such transfers, from the sample vessel (ampule or bottle) to the sample beaker, from the sample beaker to the ion exchange column and from the beaker containing the eluted sodium fraction to the platinum crucible. There are also steps where mechanical loss might occur (e.g., wet-ashing or the early stages of sample ignitions).

In the first transfer, mechanical loss or evaporation could introduce an error. Based on the potential combined mechanical and evaporative losses, the maximum relative uncertainty for serum samples is estimated to be 0.040 % and the maximum relative uncertainty for samples of NaCl is estimated to be 0.020 %.

The sources of systematic error and uncertainty for the other two transfers were easy to evaluate since all of the beakers and crucibles associated with a sample could be extracted with 1 mol/L HNO_3,_ to remove any residual Na. These extracts were analyzed by FAES. Residual sodium levels of 2 μg to 3 μg were found, much of which was probably measurement blank. At worst, this transfer has a maximum uncertainty of 3 μg or 0.019 % for an average serum sample yielding 50 mg of Na_2_SO_4_.

Mechanical loss due to splatter is also a potential problem although the samples were covered during ignitions and wet digestion. Furthermore, during the evaporation steps after digestion or column elution, losses could occur. The beaker and the cover were examined after the transfer steps in the experiment by washing their surfaces with 1 mol/L HNO_3_ and determining Na by FAES. To make the experiment more meaningful, only the lower half of each beaker was rinsed with water during the transfer process. Thus the experiment was designed to determine if any Na was on the upper half of the beaker wall. The amounts detected were similar to those in the prior experiment, only 1 μg to 3 μg of Na found. At worst, this transfer has a maximum uncertainty of 3 μg or 0.019 % for an average serum sample.

The loss of sample in the transfer from the sample beaker to the ion exchange column or from the beaker containing the eluted sodium fractions to the platinum crucible is not likely, although possible. The loss of a 2 μL droplet, which is seen easily, is equivalent to 0.007 % of the sample for transfer of 30 mL to the column and 0.025 % of the sample for transfer of 8 mL to the crucible. A competent analyst can transfer the samples quantitatively to the columns.

The maximum systematic error from all of these potential losses would be the linear sum of these losses. The systematic error caused by these losses would be somewhere in the interval of zero to the maximum sum. The magnitude of the correction to compensate for this systematic error is estimated using the assumption that it is most likely that there is not an error and that the probability of error linearly decreases to zero at the maximum relative error. Therefore, the mean error can be calculated [12] as having a magnitude of one-third of the maximum interval. This error has the effect of decreasing the apparent sodium value. The negative of this error is added as a correction in terms of the equivalent mass of Na, on a sample-to-sample basis. For the average serum the linear sum of the potential errors from transfer losses equals 0.109 %. The mean error is one-third this value or –0.036 %. The relative standard uncertainty is calculated [12] by multiplying the magnitude of the correction by $\sqrt{3}$. For the average serum the relative standard uncertainty equals 0.063 %.

### 3.2 Density

Since all the measurements are based upon gravimetric sampling, one significant uncertainty in sampling is in measuring the serum’s density, which is used to change results from mass divided by mass to mass divided by volume. This measurement usually results in a relative standard uncertainty of 0.02 % for serum samples. This uncertainty is used to calculate the sample volume uncertainty.

### 3.3 Sample Mass

The sample mass was determined to a resolution of 1 mg and was relative to a tare with a resolution of 1 mg. The uncertainty of this mass is determined by summing in quadrature which would equal 1.4 mg. The sample mass was determined as the difference between the mass of a full syringe or pipette and the mass of the empty syringe or pipette so that the total uncertainty is determined by summing 1.4 mg in quadrature to yield 2 mg. This value is converted to a standard uncertainty by dividing by $\sqrt{3}$ to equal 1.2 mg. The relative standard uncertainty of the sample mass is calculated by dividing the 1.2 mg standard uncertainty by the mean sample mass. For the average serum the relative standard uncertainty equals 0.021 %. This uncertainty is used to calculate the sample volume uncertainty.

The sample mass for SRM 919a was determined to a resolution of 2 μg which has a standard uncertainty of 2.4 μg. The relative standard uncertainty is calculated by dividing by the mean mass of 140 mg to equal 0.002 % for SRM 919a.

### 3.4 Sample Volume

The sample volume is calculated by dividing the sample mass by the serum density. The relative standard uncertainty of the volume is calculated by adding the standard uncertainties of the mass and density in quadrature. For the average serum this relative standard uncertainty equals 0.029 %.

### 3.5 Ion Exchange Chromatography

There are several potential sources of systematic error that may occur in the chromatographic separation and purification phases of the experiment. The quantitative absorption of Na and the selective elution of Na from other cations must be complete to avoid analytical errors.

The retention of Na on the column was evaluated by examination of the sample washings up to the elution of Na itself. In addition, the column was stripped with ~5 mol/L HCl after the Na fraction was eluted to evaluate the extent to which Na remained on the column. These fractions and washings were concentrated to remove excess acid and the Na was determined. The columns were scaled to accept ~50 mg of Na along with the other usual serum constituents. The only possibility for loss of Na on column loading would be because of channelling through the resin bed. The experiments indicated that a total of less than 5 μg of Na might be lost through sample loading or retention on the column after elution of the Na fraction. Again, this Na could be a blank and represents an upper limit based upon the reproducibility of blank measurements in Pt crucibles. A maximum systematic error of 5 μg is estimated for sample loading or retention on the column. The mean error, correction, and uncertainty are calculated in the same manner as the transfer losses. For the average serum the mean relative correction equals 0.010 % and the relative standard uncertainty equals 0.018 %.

The issue of elemental purity of the eluted sodium fraction is more complex. All of the likely impurities (Li, K, Ca, Mg) form sulfates and could cause a corresponding gravimetric error. In addition, some sodium is lost to the Li and K fractions. For serum samples that were added directly to the column, the protein cake that occasionally formed at the top of the column caused band broadening, which resulted in poorer separation of Na from Li and K. When the samples were wet-digested, no sodium was detected in the lithium and potassium fraction because better separation of the sodium was achieved. Since some tailing of the elements can occur as they elute from the column, the fraction collected for Na can vary slightly from sample to sample. There were some samples that had poor separation between elements as indicated by flame tests in which sodium was detected in either the lithium or potassium fraction. These samples which had a deficiency in the separation were tested by analyzing the Li and K fractions for Na and the Na fraction for Li, K, Ca, Mg, and Fe (Fe contamination of the Na_2_SO_4_ resulted from iron contamination of certain platinum crucibles and was observable as a dark stain inside the crucible). These fractions were measured for volume and their constituents determined by FAAS or FAES. The results were converted to a total equivalent mass of sodium as the sulfate [10] and are summarized in Tables 1 and 2. The errors are partially compensating in this part of the analysis. That is, when the Na fraction was contaminated by other elements, other fractions showed sodium as a contaminant. In samples with observed poor separation, the sodium losses (as Na_2_SO_4_) to other fractions were as much as 8.5 mg (up to a maximum of 0.014 % of the mass as Na_2_SO_4_), while the elements (as sulfates) found in the sodium fraction could add up to 57 μg to the mass of the precipitate (up to a maximum of 0.098 % of the mass as Na_2_SO_4_). Corrections were made for these losses or contaminants based on the associated FAAS or FAES measurements. The estimated maximum uncertainty of the instrumental determinations is 5 %, which would give a maximum relative uncertainty of 0.001 % for the sodium loss correction and 0.005 % for the contamination correction. If the flame tests did not show an overlapping of the fractions, no corrections were made. Based on the sensitivity of the flame tests, the estimated uncertainty of assuming no losses or contamination is no more than 0.006 %. A maximum uncertainty of 0.006 % to cover all cases (with and without contaminations) is used. This maximum uncertainty most closely represents a confidence level of 99 % (for an assumed normal distribution) and is divided it by 2.576 to obtain a relative standard uncertainty of 0.002 % [11].

### 3.6 Influence of Sample Form on Ion Exchange

In the original method, serum was diluted with distilled water and applied directly to the ion exchange columns [2]. This procedure was modified by wet-ashing the serum to eliminate the occasional column blockage caused by the precipitation of the serum protein. This clogging severely retards the elution rate.

As already established above, no significant amount of Na was retained on the column after elution of Na. A comparison of both methods of sample addition to the column on the same sample types did not reveal any significant differences between the final mean results.

### 3.7 Blanks

The contribution of the analytical blank for Na to the uncertainty of the analysis was evaluated in two ways. By extraction and direct analysis, the total amount of Na extractable from the beakers, columns, and platinum crucibles was found to be typically less than 1 μg to 2 μg. This amount is trivial relative to other possible sources of error. In the second method, empty crucibles were used as weighing tares to compensate for changes in mass resulting from changes in barometric pressure, relative humidity, or temperature. The tares were also the blank samples, so that any blank contribution was corrected also. Thus, if tare crucibles increased in mass by 10 μg, then 10 μg was subtracted from the apparent mass of the sample crucibles. Any individual crucible mass carries an uncertainty of 5 μg to 10 μg. Since the contribution of the blank to the uncertainty is negligible compared to changes in tare mass and uncertainties in the crucible mass, it is factored into the estimate of uncertainty for the weighing and ignition of the samples.

### 3.8 Ignition and Weighing of Samples

The greatest difficulty in the analysis is the attainment of a stable mass of the Na as Na_2_SO_4_. One complication is because Na tends to form NaHSO_4_ [7, 13] and its conversion to Na_2_SO_4_ is slow. The addition of H_2_O or (NH_4_)_2_CO_3_ between ignitions accelerates this conversion and the Na blank in the (NH_4_)_2_CO_3_ is insignificant. Elevating the temperature of the ignition also assists in the conversion to Na_2_SO_4_, but higher temperatures will cause loss of Na through the volatilization of Na_2_SO_4_. The conditions described for the ignition represent a compromise between loss and incomplete conversion. The amounts of Na volatilized are small, but measurable.

In addition, Na_2_SO_4_ is somewhat hygroscopic which leads to complications in weighing the crucibles without significant error. On the most humid days of the summer, it may not be possible to reproduce sample mass from ignition to ignition because of the hygroscopic properties of Na_2_SO_4_.

Sample ignitions are not expected to lose Na at a rate of more than 10 μg/h at 900 °C. Since it is difficult to reproduce weighings to better than ±5 μg, 20 μg agreement was chosen as the decision level for constant mass after successive ignitions. If the difference between successive ignitions was greater than 20 μg, the ignition was repeated. Standards and samples exhibited the same kind of behavior under the conditions used for ignition.

By summing the observed variability in tare mass, sample mass, and loss on ignition, an uncertainty of 50 μg (as Na_2_SO_4_) in the weighing and ignition of samples is estimated. Since this estimate most closely follows a 95 % confidence interval (of an assumed normal distribution) it is corrected to a relative standard uncertainty by dividing by 1.96 to obtain 26 μg. For the average serum this corresponds to a relative standard uncertainty of 0.052 %. Moisture absorbed during sample weighings could add as much as another 50 μg uncertainty when relative humidity conditions are greater than 60 %.

### 3.9 Recovery of Standards

After all allowances are made for potential sources of systematic error, it is common practice to test a method for error with a known standard. A NaCl assay standard (SRM 919) was used for this purpose, where the assay of this SRM was based on Cl and stoichiometry was assumed. Based upon a consideration of the impurities present, the certificate for SRM 919 gives a purity of 99.9 % as NaCl.

For evaluating the accuracy of the definitive method a maximum uncertainty of less than 0.1 % was estimated for the Na concentration in a prepared standard, which was a mixture of NaCl (SRM 919) and KCl (SRM 999) to simulate natural serum levels for Na and K. This uncertainty covers both the uncertainties in the solution preparation. Over the ~15 year period that the definitive method has been employed, assays of the standard mixture always have given an apparent Na recovery of greater than 99.9 % with a standard deviation of 0.15 %, which would be equivalent to a standard deviation of the mean of 0.075 % for four samples. These results demonstrate both the accuracy and repeatability of the technique. A relative standard uncertainty of 0.075 % is used for the recovery of the sodium standard for serum. For SRM 919a a relative standard uncertainty of 0.019 % is used based on the results that follow.

### 3.10 Recent Improvements and Observations

The opportunity to assay Na in a new SRM NaCl (SRM 919a) provided an opportunity to evaluate the ignition and weighing errors under more optimum conditions. Since the NaCl was of high purity and was dissolved in water and poured directly onto the column, uncertainties of sample transfer, sample digestion and elemental separations could be avoided. The results given in Table 3 are therefore representative of the minimum uncertainty that might be expected from the ion exchange-gravimetric method. The high degree of repeatability obtained using an average sample mass of ~170 mg as Na_2_SO_4_ suggests that the problems found for the analysis of Na in serum are caused primarily by inherent limitations in the measurement of a small amount (~50 mg) of Na_2_SO_4_ in a crucible with a mass of ~20 g. The accuracy of this method is corroborated by comparison to the coulometric determination of chloride in SRM 919a in the assay of purity of SRM 919a as NaCl. An assay of purity of 99.891 % with a standard deviation of the mean of 0.007 % obtained by the ion exchange-gravimetric determination of sodium is in excellent agreement with an assay of 99.882 % with a standard deviation of the mean of 0.004 % based on the coulometric determination of chloride [14]. The agreement of these values also supports the assumption of a stoichiometric 1:1 ratio of NaCl in SRM 919a.

Another recent example of the application of the definitive method [2] is given in Tables 4 and 5. These results are for Na in SRM 956 (Human Serum) and SRM 909a (Freeze-dried Human Serum). One should remember that the essential difference between a definitive method and any other method is that of accuracy. Although this method is not one of the most repeatable of definitive methods, it does meet all of the essential requirements of a definitive method since sources of systematic error have been evaluated.

The uncertainties associated with the ion exchange chromatography for the recent analysis of Na in NaCl (SRM 919a) and serum (SRM 956 and SRM 909a) are slightly different than previous serum analyses. To minimize any possibility of contamination of the sodium fraction, the solution at the very beginning and very end of the sodium fraction (as determined by a flame test of a sample run concurrently) was collected for FAES determination of sodium and potassium. Total potassium (as a sulfate) was always less than 0.001 % relative to the total Na_2_SO_4_. Sodium found in these fractions is reported in Table 6. These sodium values are used to make corrections on a sample-by-sample basis. The relative magnitude of the mean correction for FAES determined Na is calculated for each sample type by dividing the mean correction (as Na_2_SO_4_) by the mean total mass of Na_2_SO_4_. For example, with SRM 919a a mean correction of 0.033 mg (Table 6) is divided by a mean mass of 170 mg to give a mean relative correction of 0.018 %. Since the relative maximum uncertainty of the instrumental determination of sodium is estimated to be 5 %, the maximum uncertainty of these corrections is only 5 % of the mean correction. Thus, for example, the estimated maximum uncertainty for SRM 919a is 0.001 % (5 % of 0.018 %). Flame tests done prior to and after the sodium fraction did not indicate any contamination by Li or K. Since no contaminants were detected using the flame tests and FAES, the Na_2_SO_4_ was not analyzed for contaminants. The uncertainty associated with potential contaminants in the Na_2_SO_4_ is the same as discussed previously for the general case.

The recovery of SRM 919, NaCl, run as a control, was quite good also for these recent determinations. For the determination of sodium in SRM 919a, 4 control samples of SRM 919 yielded an average recovery of 99.988 % relative to the certified value with a standard deviation of the mean of 0.019 %. For the determination of sodium in SRM 956, 4 control samples of SRM 919 yielded an average recovery of 99.972 % relative to the certified value with a standard deviation of the mean of 0.067 %. For the determination of sodium in SRM 909a, 2 control samples of SRM 919 yielded an average recovery of 100.029 % relative to the certified value with a standard deviation of the mean of 0.031 %. In addition, since the source material for SRM 919a was a much purer source of sodium than serum, the relative standard uncertainty associated with potential contaminants in the Na_2_SO_4_ for SRM 919a is estimated to be half that of the general case or 0.002 %/2 = 0.001 % [11].

### 3.11 Summary of Sources of Uncertainty

The sources of uncertainty and calculated values for selected recent samples and a typical serum sample (50 mg as Na_2_SO_4_) from previous analyses [2] are listed in Table 7. The magnitude of uncertainty for the recent serum samples is based on average sample sizes (as Na_2_SO_4_) of ~170 mg for SRM 919a, ~100 mg for SRM 956 (~10 mL of serum), and ~30 mg for SRM 909a (~3 mL of serum). All standard uncertainties are calculated using the CIPM approach [11].

Table 8 provides a summary of the calculated uncertainty values for recent samples and a typical serum sample based on data and uncertainty from previous analyses [2]. The only sources of Type A uncertainty are the measurement repeatability and the recovery of standards, which are both expressed as the standard deviation of the mean. These uncertainties were combined as the square root of the sum of the squares for the combined Type A uncertainty. The sources of Type B uncertainty are the uncertainties of sample handling, sample volume (calculated from density and sample mass), ion exchange chromatography, and sample weighing and ignition. These uncertainties were combined as the square root of the sum of the squares for the combined Type B uncertainty. The expanded uncertainty is calculated as the square root of the sum of the squares of the combined Type A and combined Type B uncertainties and multiplied by a coverage factor based on the equivalent degrees of freedom. The relative expanded uncertainty is 0.09 % for SRM 919a, 0.24 % to 0.45 % for SRM 956, 0.27 % to 0.37 % for SRM 909a, and 0.64 % for a typical serum sample using the previously estimated uncertainties [2] which have been recalculated using the CIPM approach.

## 4. Conclusion

After some 15 years of usage at NIST by two generations of chemists, a considerable body of data exists on the usefulness of this definitive method for determining sodium in serum. Reasonable care has been taken to estimate and evaluate systematic sources of measurement error and uncertainty. The recovery of sodium from standards (SRM 919, NaCl) has always been within 0.15 % of the theoretical value. Although this is not an extremely repeatable method, the sources of uncertainty in the method are well understood. Better repeatability was obtained when the same procedure was utilized for the analysis of Na in SRM 919a, NaCl. This result tends to suggest that most of the uncertainty in the definitive method arises from the sample handling of the serum, the small sample size of serum, the low concentration of sodium, and the subsequent small mass of Na_2_SO_4_ produced.

The sum of all uncertainties for the definitive method has been said to be ~0.75 % when a different approach to calculating uncertainty was used [2]. This uncertainty has been recalculated using the CIPM approach as a relative expanded uncertainty equal to 0.64 %. Review of a considerable number of data sets shows that the uncertainty can vary as function of the number of samples taken, the mass of the sample taken, and whether or not the sample is freeze-dried. Based on all of these data sets and our improved repeatability, we now estimate the relative expanded uncertainties for serum samples to be 0.3 % to 0.4 %.

We consider this method for sodium to be definitive at NIST and have used it to certify serum SRMs and to validate the accuracy of the NCCLS reference method for sodium in serum (FAES) [2]. The accuracy of the FAES reference method and the NIST reference materials (SRMs 956 and 909a) are closely linked to this definitive method for sodium. We do not advocate its routine use in the clinical lab. However, the significance of this definitive method should be recognized in the metrological hierarchy by which routine clinical laboratory results are tied to national standards.

## Figures and Tables

**Table 1:** Samples with poor separation: loss of sodium to fraction before (pre-) or after (post) Sodium Fraction

Sample	Na (μg)	Equivalent Na_2_SO_4_ (μg)	Na_2_SO_4_ precipitate mass (mg)	Relative to total Na_2_SO_4_ (%)
All pre-	< 0.1	< 0.3	75.0	< 0.0004
Post 1	0.5	1.4	61.5	0.002
Post 2	2.7	8.5	62.6	0.014
Post 3	1.6	4.8	71.3	0.007
Post 4	0.7	2.2	64.0	0.003

**Table 2:** Contamination of the sodium fraction in samples with poor separation

Sample	Li (μg)	K (μg)	Ca (μg)	Mg (μg)	Fe (μg)	Total as sulfate (μg)	Na_2_SO_4_ precipitate mass (mg)	Relative to total Na_2_SO_4_ (%)^b^	Total^a^ as sulfate (μg)	Relative to total Na_2_SO_4_ (%)^a^,^b^
1	0.1	3.3				8	90	0.009	8	0.009
2	3.0	4.0				33	83	0.040	33	0.040
3	1.2	2.0				14	80	0.017	14	0.017
4	0.0	1.2				3	80	0.004	3	0.004
5	1.8	1.7				18	100	0.018	18	0.018
6	1.5	1.9				16	80	0.020	16	0.020
7		6.4	1.6	0.6	3.0	33	73	0.046	30	0.042
8		16.4	3.1	0.4	3.0	60	58	0.103	57	0.098
9		6.8	0.5	0.2	59.0	229	58	0.396	170	0.294^c^
10		1.0	0.6	0.3	2.0	13	76	0.017	11	0.014
11		3.0	0.3	0.2	5.0	27	73	0.036	22	0.029
12					< 0.1	0.1	20	< 0.001	0.1	< 0.001
13					0.8	3	65	0.005	2	0.003
14					< 0.1	0.1	20	< 0.001	0.1	< 0.001

a Corrected by subtraction of metallic Fe assumed present in empty Pt crucible before sample addition.

b Calculated as the mass of the sulfate contaminants relative to the total gravimetric mass.

c This sample had a very dark stain of iron in the crucible and was not considered in calculations.

**Table 3:** Assay of purity of NaCl (clinical standard) SRM 919a based on sodium determination

	Purity (as NaCl) (%)	Mass fraction sodium (%)
	99.887	39.293
	99.916	39.304
	99.888	39.293
	99.850	39.278
	99.895	39.296
	99.915	39.304
	99.888	39.293
	99.904	39.300
	99.900	39.298
	99.906	39.300
	99.851	39.279

Mean value	99.891	39.294
Expanded uncertainty	0.089	0.029

**Table 4:** Sodium in human serum, SRM 956

	Level I (mmol/L)	Level II (mmol/L)	Level III (mmol/L)
	122.25	141.56	161.33
	122.11	142.22	161.38
	122.17	141.24	161.10
	122.04	141.30	161.06
	122.09	142.01	

Mean value	122.13	141.67	161.22
Expanded uncertainty	0.39	0.64	0.39

**Table 5:** Sodium in freeze-dried human serum, SRM 909a

	Level I (mmol/L)	Level II (mmol/L)
	148.70	127.27
	148.54	126.54
	148.05	126.52
	148.38	126.32
	148.82	126.31
	148.79	126.15

Mean value	148.54	126.52
Expanded uncertainty	0.55	0.35

**Table 6:** Total sodium found in solutions collected at front and back tails of the sodium fraction

SRM	Sample	FAES Na (μg)	Na as Na_2_SO_4_ (μg)	Total Na_2_SO_4_ (mg)	Relative to total Na_2_SO_4_ (%)
919a	1	9.0	28	158	0.018
	2	8.5	26	216	0.012
	3	24.0	74	206	0.036
	4	16.6	51	209	0.025
	5	28.9	89	202	0.044
	6	4.2	13	128	0.010
	7	1.6	5	106	0.005
	8	5.4	17	193	0.009
	9	2.2	7	136	0.005
	10	7.1	22	160	0.014
	11	10.9	34	157	0.021

	**mean**	**10.8**	**33**	**170**	**0.018**

956	I-1	3.3	10	26	0.039
	I-2	2.3	7	26	0.027
	I-3	3.3	10	26	0.039
	I-4	0.4	1	26	0.005
	I-5	0.5	2	26	0.006

	**mean**	**2.0**	**6**	**26**	**0.023**

	II-1	3.2	10	30	0.032
	II-2	2.5	8	31	0.025
	II-3	1.2	4	31	0.012
	II-4	3.5	11	31	0.035
	II-5	1.9	6	31	0.019

	**mean**	**2.5**	**8**	**31**	**0.025**

	III-1	5.1	16	35	0.045
	III-2	0.7	2	36	0.006
	III-3	0.7	2	35	0.006
	III-4	0.7	2	35	0.006

	**mean**	**1.8**	**6**	**35**	**0.016**

909a	I-1	42.7	132	92	0.144
	I-2	31.3	97	91	0.106
	I-3	27.1	84	93	0.090
	I-4	27.3	84	94	0.090
	I-5	10.2	32	93	0.034
	I-6	10.9	34	92	0.037

	**mean**	**24.9**	**77**	**92**	**0.083**

	II-1	5.8	18	107	0.017
	II-2	7.7	24	107	0.022
	II-3	6.5	20	106	0.019
	II-4	5.5	17	108	0.016
	II-5	7.7	24	108	0.022
	II-6	10.2	32	108	0.029

	**mean**	**7.2**	**22**	**107**	**0.021**

**Table 7:** Calculated relative standard uncertainties for selected samples

Uncertainty type	Source of uncertainty	Relative standard uncertainties			Previous typical serum^b^ (%)
		SRM 919a^a^ (%)	SRM 956 Level I (%)	SRM 909a Level I (%)	
A	Measurement replication	0.007	0.029	0.081	0.219
A	Recovery of standards	0.019	0.075	0.075	0.075
B	Sample handling	0.036	0.082	0.053	0.063
B	Sample volume	0.002	0.044	0.023	0.029
	Ion exchange chromatography				
B	loading or retention	0.005	0.034	0.010	0.018
B	loss or contamination	0.001	0.002	0.002	0.002
B	Weighing and ignition	0.015	0.099	0.028	0.052

a Certain uncertainty values for SRM 919a are different from the serum as explained in text.

b Velapoldi et. al. [2]

**Table 8:** Summary of relative uncertainties

Uncertainty	SRM 919a	SRM 956 Level I	SRM 956 Level II	SRM 956 Level III	SRM 909a Level I	SRM 909a Level II	Previous typical serum
Combined Type A	0.020 %	0.080 %	0.162 %	0.091 %	0.110 %	0.148 %	0.231 %
Combined Type B	0.040 %	0.140 %	0.140 %	0.065 %	0.065 %	0.088 %	0.088 %
Combined relative standard uncertainty	0.045 %	0.161 %	0.214 %	0.112 %	0.128 %	0.172 %	0.248 %
Effective degrees of freedom	> 30	> 30	18	12	14	14	5
Coverage factor^a^	2	2	2.1	2.18	2.14	2.14	2.57
Relative expanded uncertainty	0.089 %	0.323 %	0.449 %	0.244 %	0.274 %	0.369 %	0.636 %

a Coverage factor equals 2 unless degrees of freedom < 30 [11].

## Footnotes

1 Certain commercial equipment, instruments, or materials are identified in this paper to foster understanding. Such identification does not imply recommendation or endorsement by the National Institute of Standards and Technology, nor does it imply that the materials or equipment identified are necessarily the best available for the purpose.
